# Environmental risk factors associated with community diarrheal disease in Ethiopia

**DOI:** 10.1186/s12889-025-23086-4

**Published:** 2025-05-27

**Authors:** Devin LaPolt, Sage Smith, Lina Gazu, Silvia Alonso, Amete Mihret Teshale, Binyam Moges Azmeraye, Galana Mamo Ayana, Dessie Abebaw Angaw, Desalegne Degefaw, Ariel V. Garsow, Aaron Beczkiewicz, Getnet Yimer, Michala J. Krakowski, Robert Scharff, Eyasu T. Seyoum, Barbara Kowalcyk

**Affiliations:** 1 Department of Food Science and Technology, The Ohio State University, Columbus, OH USA; 2 College of Public Health, The Ohio State University, Columbus, OH USA; 3 Health Program, International Livestock Research Institute, Addis Ababa, Ethiopia; 4 National Clinical Bacteriology and Mycology Reference Laboratory, Ethiopian Public Health Institute, Addis Ababa, Ethiopia; 5 The Ohio State Global One Health Initiative Eastern Africa Regional Office, Addis Ababa, Ethiopia; 6 Department of Epidemiology and Biostatistics, School of Public Health, College of Health and Medical Science, Haramaya University, Harar, Ethiopia; 7 Department of Epidemiology and Biostatistics, College of Medicine and Health Sciences, School of Public Health, University of Gondar, Gondar, Ethiopia; 8 Department of Food Science and Technology, Center for Foodborne Illness Research and Prevention, The Ohio State University, Columbus, OH USA; 9 Department of Genetics, Penn Center for Global Genomics & Health Equity, Perelman School of Medicine, University of Pennsylvania, Philadelphia, PA USA; 10 Department of Human Sciences, The Ohio State University, Columbus, OH USA; 11 Translational Data Analytics Institute, The Ohio State University, Columbus, OH USA; 12 Milken Institute School of Public Health, George Washington University, Washington, DC USA

**Keywords:** Diarrheal diseases, Environmental health, WASH, Ethiopia

## Abstract

**Background:**

Diarrhea is a major contributor to mortality in sub-Saharan Africa, where access to improved sanitation and clean water is limited. Identifying factors associated with diarrhea across geographical regions and age groups can inform resource allocation toward water infrastructure, healthcare, and disease mitigation. The objective of this study was to identify environmental risk factors associated with diarrhea in the general population across multiple communities in Ethiopia.

**Methods:**

A prospective cross-sectional study was conducted in three regions in Ethiopia from October 2021-October 2022. REDCAP mobile app was used to collect data during face-to-face interviews using a structured, pretested questionnaire. Descriptive statistics characterized household environmental exposures. Univariate and multivariable logistic regression were used to identify factors associated with diarrhea.

**Results:**

A total of 2,436 households participated in the study. Of these, 10.3% of households reported at least one case of diarrhea during the previous four weeks. Household animal ownership varied by site, with Addis Ababa reporting the lowest animal ownership and Harar reporting the highest ownership. Across all sites, pit latrines without covers were the most common sanitation facility. Water piped to yard was the most frequent source of drinking water and most households did not use treated water (boiled/filtered) when handling food. No environmental factors were associated with diarrhea in Addis Ababa. In Gondar, drinking water from unprotected wells was associated with increased odds of diarrhea [COR:4.81(95%CI:2.03,11.43)]. Dry season was associated with decreased odds of diarrhea compared to short [COR:0.42(95%CI:0.24,0.75)] and long rains seasons [COR:0.55(95%CI: 0.34,0.88)]. In Harar, drinking water from communal taps was associated with increased odds of diarrhea [COR:2.02(95%CI:1.32,3.09)].

**Conclusion:**

Multiple environmental factors for diarrhea were identified. Given the variation in factors by site, strategies for intervention and management should be community-specific. These factors can be managed through improved water treatment, sanitation practices, and educational programs focused on proper hygiene. Efforts to manage these factors can potentially reduce the burden of diarrheal diseases.

**Supplementary Information:**

The online version contains supplementary material available at 10.1186/s12889-025-23086-4.

## Background

Diarrheal disease is a major contributor to morbidity and mortality in regions where frequent animal exposures, and inadequate water, sanitation, and hygiene (WASH) practices are common, such as sub-Saharan Africa [1–3]. For example, household contact with domesticated animals has been associated with an increased risk of diarrheal disease in multiple countries, especially in areas where sharing living space with domesticated animals is common practice [3–5]. Additionally, the use of unimproved sanitation facilities has been associated with diarrheal disease in many countries [6]. The World Health Organization (WHO) estimates that over half of all diarrheal disease mortality globally is associated with unimproved or inadequate WASH practices [7]. While efforts to improve WASH practices have improved global public health [8], in many low- and middle-income countries (LMICs), access to clean drinking water is still limited, and contaminated water is used for food preparation activities, influencing the risk of diarrhea and symptoms commonly associated with foodborne disease (FBD) [9, 10]. Additionally, since diarrhea can be attributed to both infectious (e.g., bacteria, parasites, viruses) and non-infectious (e.g., malnutrition) sources, environmental factors cannot be attributed to all cases of diarrhea in a community.

Household ownership of livestock, poultry, and other domestic animals has been previously associated with diarrheal disease in multiple LMICs, including Ethiopia. For example, a recent study in southwest Ethiopia found that children under the age of five had 2.87 times the odds of diarrhea when sharing a residence with domestic animals [11]. Similarly, a recent study conducted in northwest Ethiopia also identified households sharing space with domestic animals to have 3.3 times the odds of diarrhea for children under five [12]. Further, a study assessing the prevalence and load of *Campylobacter*, which commonly causes diarrhea, in infants in rural Ethiopia found that the pathogen was frequently identified in livestock feces and contaminated soil was found to contribute to infection [13]. To the best of our knowledge, no studies in Ethiopia have assessed the association between livestock ownership and diarrhea in children over five and adults. However, a recent study conducted in Cambodia identified an association between household poultry ownership and diarrhea [14]. Given that over 90% of households in Ethiopia own domestic animals [15], more research is needed to better understand the relationship between how animal contact and exposure to enteric pathogens.

Unimproved sanitation has been associated with diarrheal disease in many LMICs. WHO classifies unimproved sanitation as uncovered pit latrines, flush/pour-flush to elsewhere (e.g., open areas other than septic tank, sewage system, pit latrine), buckets, hanging latrines, and open defecation [16]. Additionally, improved sanitation facilities are generally considered unimproved when shared by multiple households [16]. In sub-Saharan Africa, approximately half of all households utilize unimproved sanitation facilities [17]. However, access is far more limited in Ethiopia with over 50% of households reporting use of unimproved sanitation in 2014 [18]. Use of unimproved latrine facilities has primarily been associated with childhood diarrheal disease in Ethiopia [19]; however, this may be a risk factor for adult populations as well. For example, pit latrines have been associated with enteric infections as flooding can transport oocysts and parasites, as well as other harmful pathogens to animal and human hosts [20]. Multiple studies have identified an association between parasitic infections and latrine facilities [20–22]. Given the high use of unimproved sanitation, further research is needed to explore its relationship to exposure to diarrheal disease-causing agents in communities in Ethiopia.

Consumption of untreated water or mismanagement of treated drinking water sources can contribute to diarrheal disease in both children and adults in Ethiopia. Drinking untreated water is a common practice in Ethiopia as over one-third of the population regularly consumes water from unimproved sources [23]. Water contamination has been identified as a contributor to diarrheal disease in multiple studies conducted in Ethiopia [11, 24, 25]. Additionally, treated water can be contaminated through inadequate water infrastructure and poor hygiene practices. For example, a case-control study of 55 adult cases of acute bloody diarrhea and 166 controls in Northern Ethiopia found two different water collection and storage methods (dipping to collect drinking water and drinking water stored with water for other household purposes) to be independently associated with diarrheal disease [26].

The use of untreated water for handwashing during meal preparation can increase the risk of exposure to diarrheal disease-causing agents. In LMICs like Ethiopia where access to treated water is often limited, the use of untreated water to wash hands and prepare food is a common practice [9]. For example, a 2020 study of food handlers in Addis Ababa, Ethiopia, found that lack of handwashing after using toilet facilities was associated with diarrheal disease [27]. Little information is available on water treatment practices related to food handling hygiene in Ethiopia; however, further research on exposure to untreated water through food consumption is needed to better understand its association with diarrheal disease.

Integrated approaches for diarrheal disease prevention and management are critical for reducing the burden of disease in LMICs like Ethiopia. Given that Ethiopia is extremely biodiverse with a wide range of climate patterns and elevations, communities, including those of this study, can be differently impacted by environmental factors such as rainfall or temperature [28]. Identification of the environmental factors associated with diarrheal disease can inform the implementation of interventions to reduce illness. While several studies have examined the environmental risk factors associated with diarrheal disease in Ethiopia, most have focused on children under the age of five and their caregivers. Therefore, the objective of this study was to identify environmental risk factors associated with household diarrhea in the general populations in three different communities in Ethiopia.

## Materials and methods

A cross-sectional study was conducted to identify environmental factors associated with diarrhea in households from three Ethiopian communities [Addis Ababa, Gondar, and Harar (Harar Town, East Haraghe Zone)] between October 2021 and October 2022, as described in LaPolt et al. [29]. In brief, households within the defined catchment areas of the clinical laboratories of three hospitals (Yekatit 12 Hospital, University of Gondar Comprehensive Specialized Hospital, and Hiwot Fana Comprehensive Specialized Hospital) were eligible to participate in the study. The catchment areas were defined in collaboration with health professionals within each site. Sub-cities, districts, or kebeles within each site were included in the study if they were located within the catchment areas. There were no exclusion criteria, and households were included in the study if they resided in the catchment areas. Latitude and longitude points were randomly selected from the catchment areas of each site using a random number generator. Data collectors first approached the household facing east, rotating clockwise to the nearest household until a household consented to participate in the study [29]. If they did not consent to participate, a replacement point was immediately identified, and a new household was selected. A structured, pre-tested questionnaire was administered to a respondent capable of answering for the household (i.e., household head, parent, or other participant at least 15 years of age) through face-to-face interviews conducted in local languages (either Amharic or Afaan Oromo), as applicable. Participants were asked about household demographic characteristics, cases of household diarrhea within the past four weeks, household food handling practices, and household environmental exposures.

Descriptive statistics were used to characterize household exposures and demographics. Univariate and multivariable logistic regression were performed on the household-level exposure variables to assess the relationship between household environmental exposures and diarrheal disease (Table 1). Multivariate logistic regression was used to estimate the association between individual exposures and diarrheal disease after adjusting for confounders. The primary outcome of interest was household diarrhea. Exposures assessed in this analysis included animal ownership (cattle, goats, sheep, poultry, cats/dogs), toilet/sanitation facility type, drinking water source, water treatment practices for food preparation, and season of interview. Potential confounders were identified before data analysis using directed acyclic graphs (DAGs) and included in the multivariable model based on a 10% change in exposure point estimates [30, 31]. Adjusted model fit was assessed with the Hosmer-Lemeshow goodness-of-fit test. Pairwise comparisons of categorical variables were assessed using a Bonferroni adjustment; however, results were similar, and unadjusted estimates were reported.

**Table 1:** Outcomes, exposures, and potential confounders at the household level

Characteristic	Variable
Outcome	Diarrhea (Y/N)
Exposure	Cattle ownership (Y/N)
	Goat ownership (Y/N)
	Sheep ownership (Y/N)
	Poultry ownership (Y/N)
	Cat/dog ownership (Y/N)
	Sanitation facility type (Sewer system/Septic tank/Pit latrine with cover/Pit latrine without cover/open field defecation)
	Drinking water piped to dwelling (Y/N)
	Drinking water piped to yard (Y/N)
	Drinking water from a communal tap (Y/N)
	Drinking water from a neighbor’s house (Y/N)
	Drinking water from a protected well (Y/N)
	Drinking water from an unprotected well (Y/N)
	Drinking water from a protected spring (Y/N)
	Drinking water from an unprotected spring (Y/N)
	Drinking water from a surface water source
	Drinking water from a tanker truck (Y/N)
	Water treatment^a^ – water used in handwashing (Y/N/DK)
	Water treatment^a^ – water used for ASF^b^ preparation (Y/N)
	Water treatment^a^ – water used for fruit/vegetable preparation (Y/N)
	Water treatment^a^ – water used for other food preparation (Y/N)
	Season (Dry/Short rains/Long rains)
Potential confounder	Household size (5 or less/more than 5)
	Household monthly income (< 2000/2000–4000/4000–6000/6000+)

^a^ Point-of-use boiling or filtering of household water

^b^ Animal source foods

A sample size of 2,436 was determined to provide 80% power to detect seasonal and regional differences in diarrheal prevalence at a significance level of 0.05, assuming that 20% of the population was experiencing diarrhea at a given time point [29]. REDCap electronic data capture tools hosted by The Ohio State University were used for data collection and data management [32–34]. SAS 9.4 for Windows (SAS Institute Inc., USA) was used for data cleaning and statistical analysis.

## Results

### Overview of study participant characteristics

**Fig. 1:**
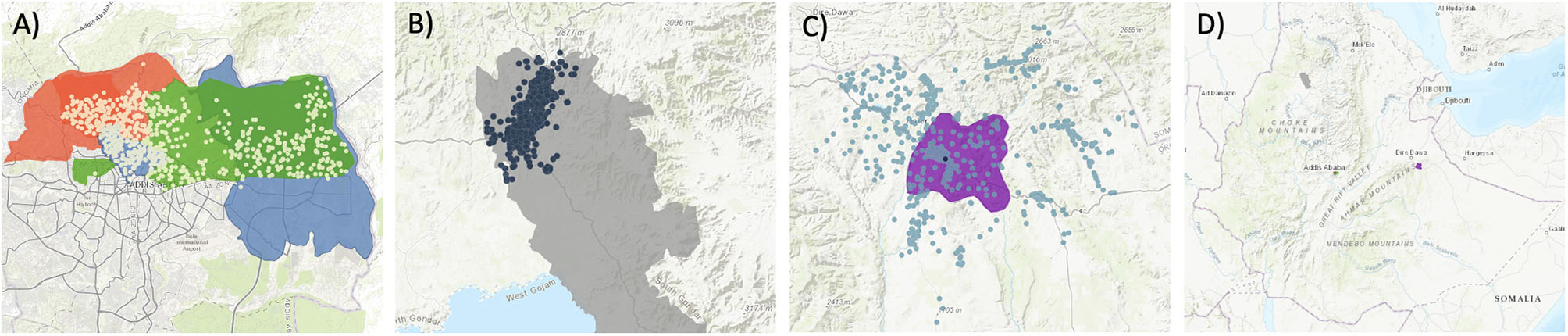
Participating households (dots) in three study sites in Ethiopia, [ArcGIS Online map hosted by Esri]. **A**) Addis Ababa. **B**) Gondar. **C**) Harar & East Hararghe. **D**) Study sites in Ethiopia

Participation of the 2,436 households enrolled in the study was evenly distributed (812/site) across study sites (Fig. 1). Of 2,436 households, most respondents were female and 20–29 years of age [29]. Household monthly income varied by site with Addis Ababa reporting a higher income than Gondar and Harar. Household size was comparable across study sites. A total of 252 (10.34%) households reported diarrhea.

### Animal ownership and occurrence of diarrheal diseases

**Fig. 2:**
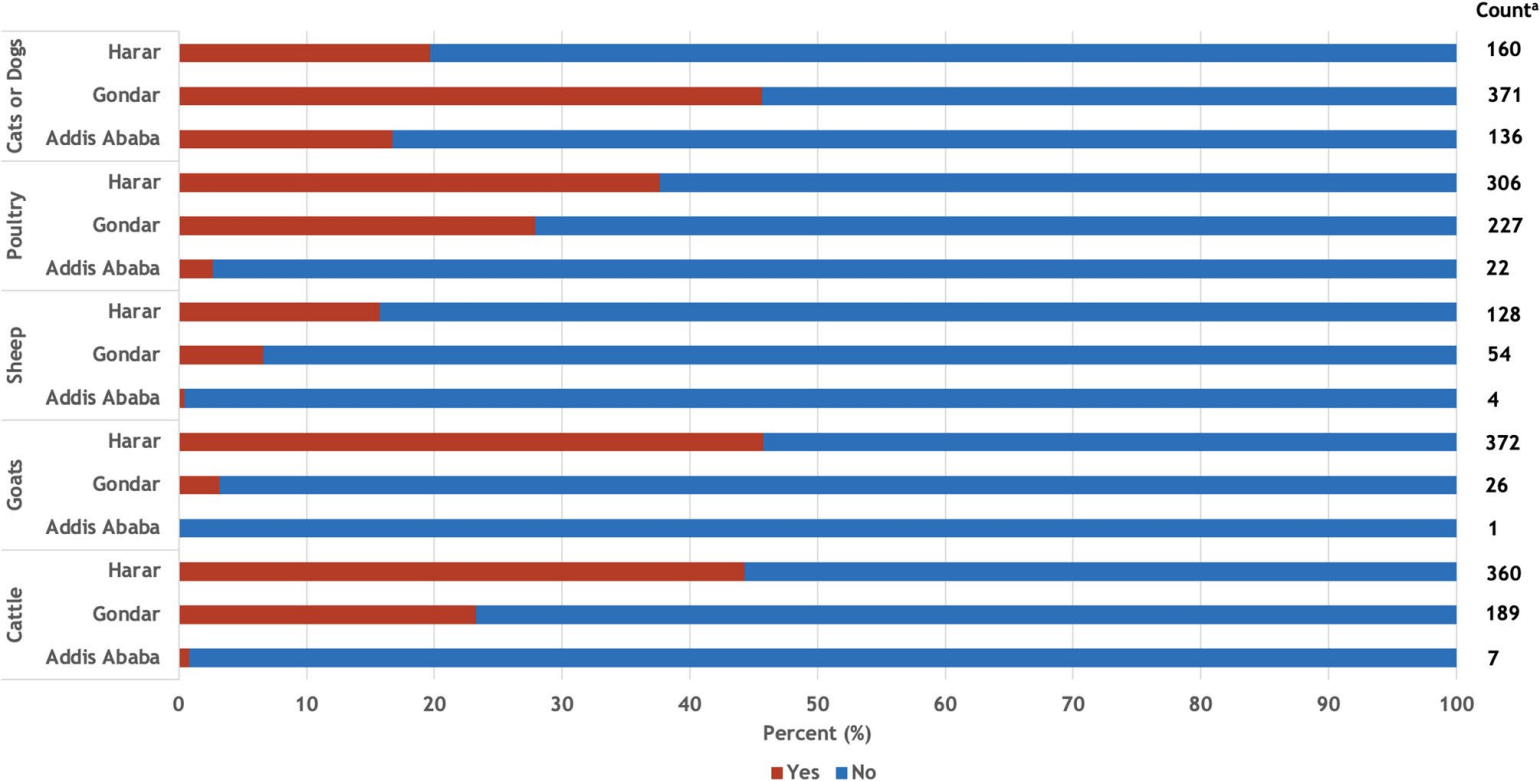
Animal ownership by study site in Ethiopia, October 2021-October 2022. ^a^ Number of households in each study site that own the respective animal

Approximately one-quarter of households reported ownership of cattle, poultry, and cats or dogs (Fig. 2). However, most households owning livestock (cattle, goats, sheep) were in Harar. Of the 252 households reporting diarrhea, animal ownership varied by study site (Table 2). For example, in Addis Ababa, none of the households with diarrhea reported ownership of livestock or poultry, while in Gondar over one-quarter of households reported livestock ownership and one-third of households reported poultry ownership. In Harar, over half of all households with diarrhea reported livestock ownership and nearly half (44.00%) of households reported poultry ownership.

### Household sanitation facility type and occurrence of diarrheal diseases

**Fig. 3:**
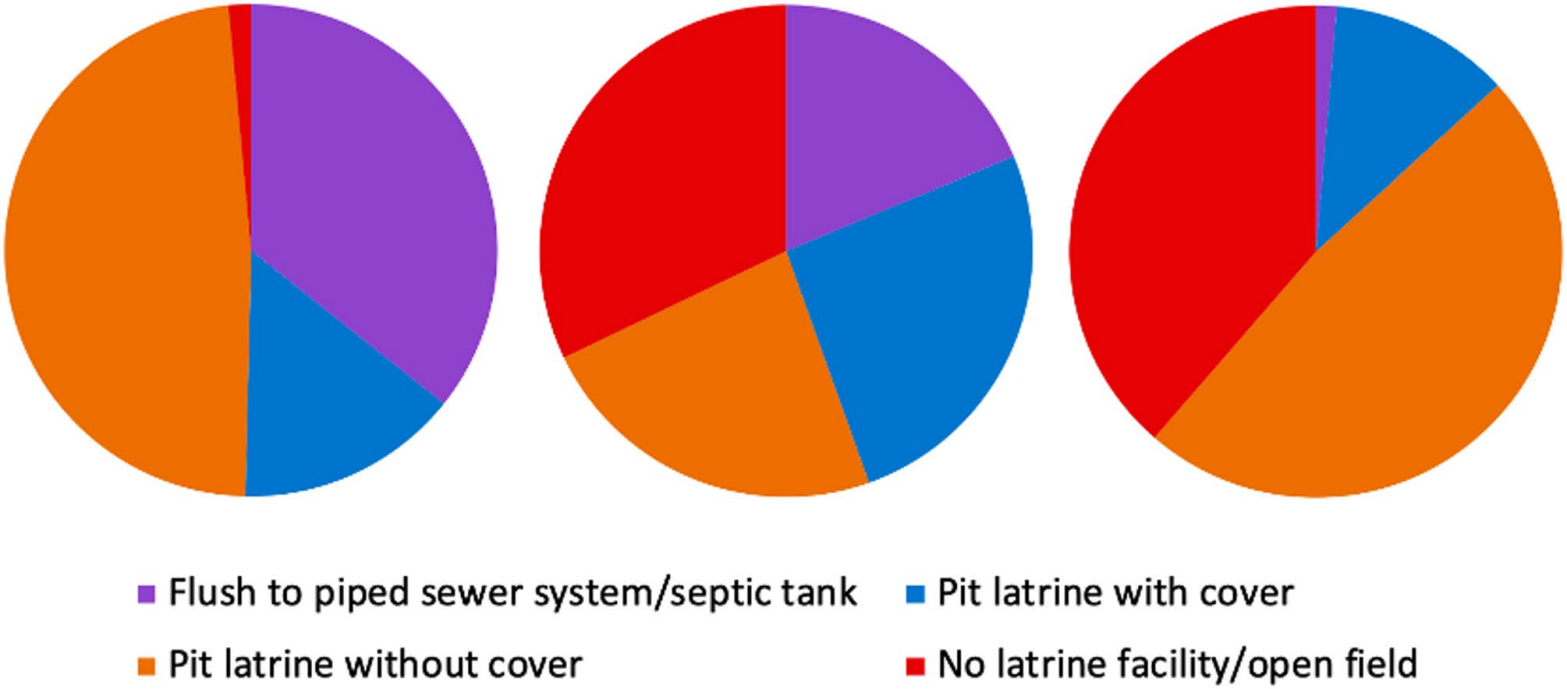
Proportion of households reporting sanitation facility type by study site in Ethiopia, October 2021-October 2022. ^A^ Addis Ababa. ^B^ Gondar. ^C^ Harar

Household sanitation facilities also varied by study site. The use of unimproved sanitation (pit latrine without cover, no latrine/open field) accounted for approximately 50% of households surveyed (Fig. 3). In Addis Ababa and Gondar, the use of improved sanitation sources such as flush-to-piped sewer systems or septic tanks and pit latrines with cover accounted for around 50% of households while in Harar, the use of improved sanitation was far less common (13.18%). Overall, the most common type of sanitation facility was a pit latrine without a cover; however, the frequency of open-field defecation was slightly higher in Harar. Most households with diarrhea (75.80%) reported the use of unimproved sanitation facilities. Of households with diarrhea, unimproved sanitation facility use varied by study site, with Addis Ababa reporting the lowest use (64.00%) and Harar reporting the highest use (93.60%).

### Drinking water source type and occurrence of diarrheal diseases

**Fig. 4:**
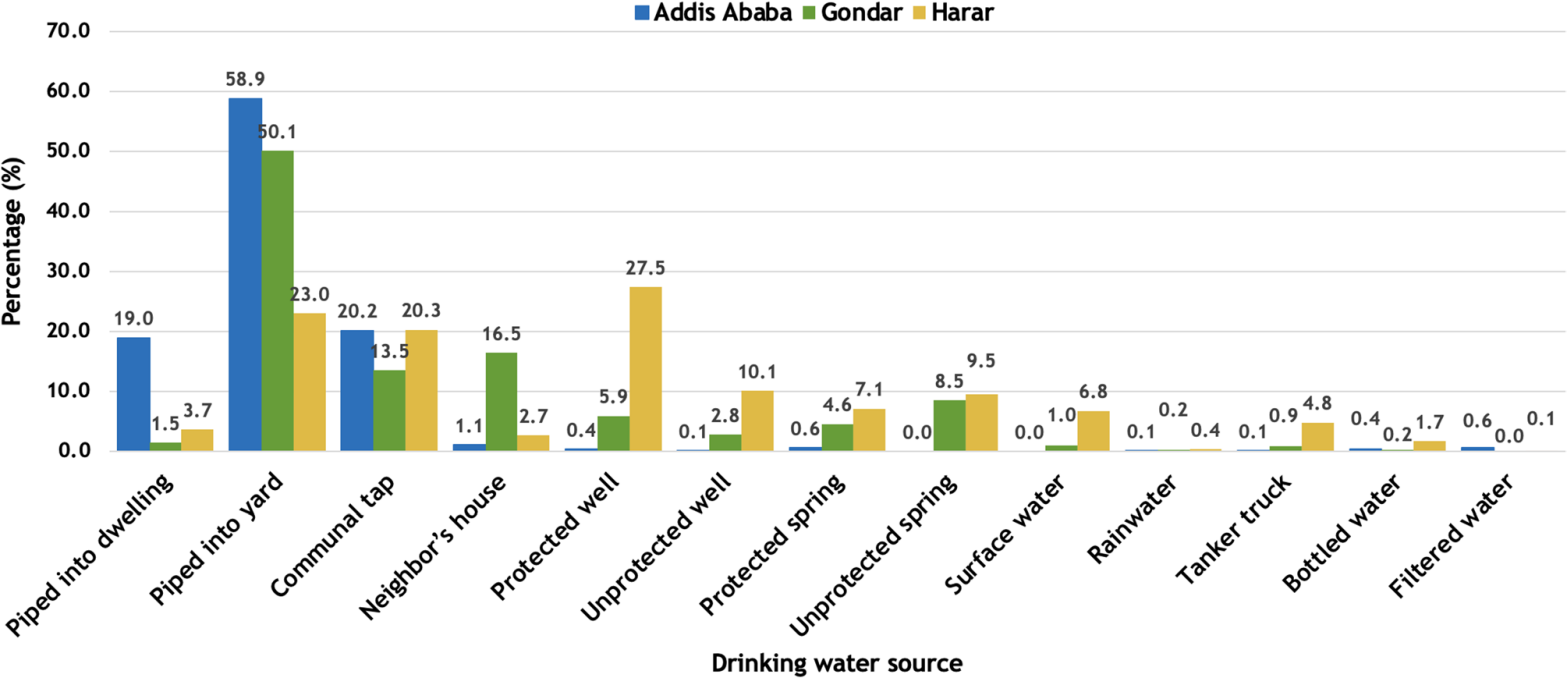
Proportion of households reporting drinking water sources by study site in Ethiopia, October 2021-October 2022

In all communities, the majority of households reported receiving drinking water from a source piped into the yard, except Harar, where protected wells were the most common water source (Fig. 4). Overall, the most common drinking water sources in households with diarrhea were water piped to the yard (30.16%) and water from a communal tap (22.62%).

### Water treatment and occurrence of diarrheal diseases

**Fig. 5:**
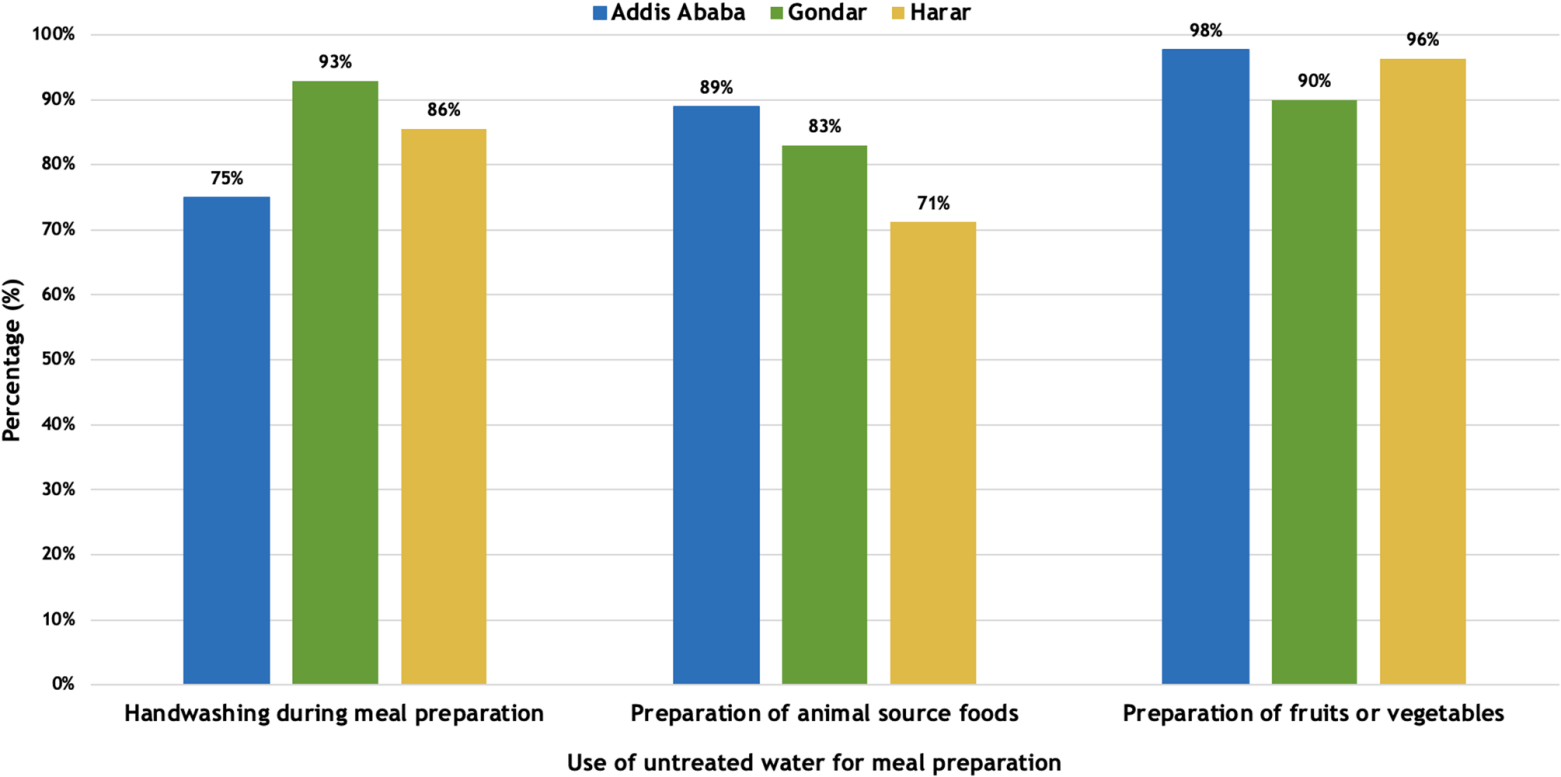
Proportion of households reporting use of untreated water during food preparation activities by study site in Ethiopia, October 2021-October 2022

Most households in all three communities reported using untreated water for handwashing when preparing food (Fig. 5). Additionally, 81.03% of all households used untreated water when preparing foods with animal-source products. Similarly, the use of untreated water for handwashing during meal preparation was frequent, ranging from 75.00% in Addis Ababa to 92.86% in Gondar, while the use of untreated water for the preparation of fruits and vegetables was over 94.62% across all study sites.

Of households reporting diarrhea, many households reported the use of untreated water for handwashing during meal preparation (92.86%), while the use of untreated water for preparation of animal source food products was lower (78.17%) (Table 2). A large number of households reported using untreated water for the preparation of meals with fruits and vegetables (92.86%) and the use of untreated water (boiled or filtered) for all food production varied greatly by site, ranging from 68.00% in Harar to 100.00% in Gondar.

**Table 2:** Community environmental exposures among households reporting diarrheal disease in three communities, Ethiopia, 2021–2022

Household characteristic		N (%)			
		Total (*n* = 252)	Addis Ababa (*n* = 25)	Gondar (*n* = 102)	Harar (*n* = 125)
Domesticated animals ^a^	Cattle ownership	90 (35.71)	0 (0.00)	26 (25.49)	64 (51.20)
	Goat ownership	77 (30.56)	0 (0.00)	5 (4.90)	72 (57.60)
	Sheep ownership	36 (14.29)	0 (0.00)	9 (8.82)	27 (21.60)
	Poultry	90 (35.71)	0 (0.00)	34 (33.33)	56 (44.80)
	Cats or dogs	77 (30.56)	7 (28.00)	48 (47.06)	22 (17.60)
Type of sanitation	Flush to piped sewer system/septic tank	20 (7.94)	5 (20.00)	14 (13.73)	1 (0.80)
	Pit latrine with cover	40 (15.87)	4 (16.00)	30 (29.41)	6 (4.80)
	Pit latrine without cover	102 (40.48)	16 (64.00)	24 (23.53)	62 (49.60)
	No latrine facility/open field	89 (35.32)	0 (0.00)	34 (33.33)	55 (44.00)
	Other	1 (0.40)	0 (0.00)	0 (0.00)	1 (0.80)
Drinking water source ^a^	Piped to dwelling	5 (1.98)	3 (12.00)	2 (1.96)	0 (0.00)
	Piped to yard	76 (30.16)	18 (72.00)	39 (38.24)	19 (15.20)
	Communal tap	57 (22.62)	3 (12.00)	15 (14.71)	39 (31.20)
	Neighbor’s house	18 (7.14)	0 (0.00)	17 (16.67)	1 (0.80)
	Protected well	35 (13.89)	0 (0.00)	6 (5.88)	29 (23.20)
	Unprotected well	22 (8.73)	0 (0.00)	9 (8.82)	13 (10.40)
	Protected spring	13 (5.16)	1 (4.00)	7 (6.86)	5 (4.00)
	Unprotected spring	26 (10.32)	0 (0.00)	9 (8.82)	17 (13.60)
	Surface water^b^	13 (5.16)	0 (0.00)	3 (2.94)	10 (8.00)
	Tanker truck	6 (2.38)	0 (0.00)	1 (0.98)	5 (4.00)
	Bottled water	4 (1.59)	0 (0.00)	1 (0.98)	3 (2.40)
	Don’t know	1 (0.40)	0 (0.00)	1 (0.98)	0 (0.00)
Untreated^c^ water uses^a^	Handwashing for food preparation	234 (92.86)	21 (84.00)	100 (98.04)	113 (90.40)
	Preparation of animal-source foods	197 (78.17)	23 (92.00)	81 (79.41)	93 (74.40)
	Preparation of fruits/vegetables	234 (92.86)	24 (96.00)	91 (89.22)	119 (95.20)
	Preparation of all foods	210 (83.33)	23 (92.00)	102 (100.00)	85 (68.00)

^a^ Some households reported more than one exposure

^b^ Rivers, canals, lakes

^c^ Water not boiled or filtered

### Environmental factors associated with diarrheal diseases

**Fig. 6:**
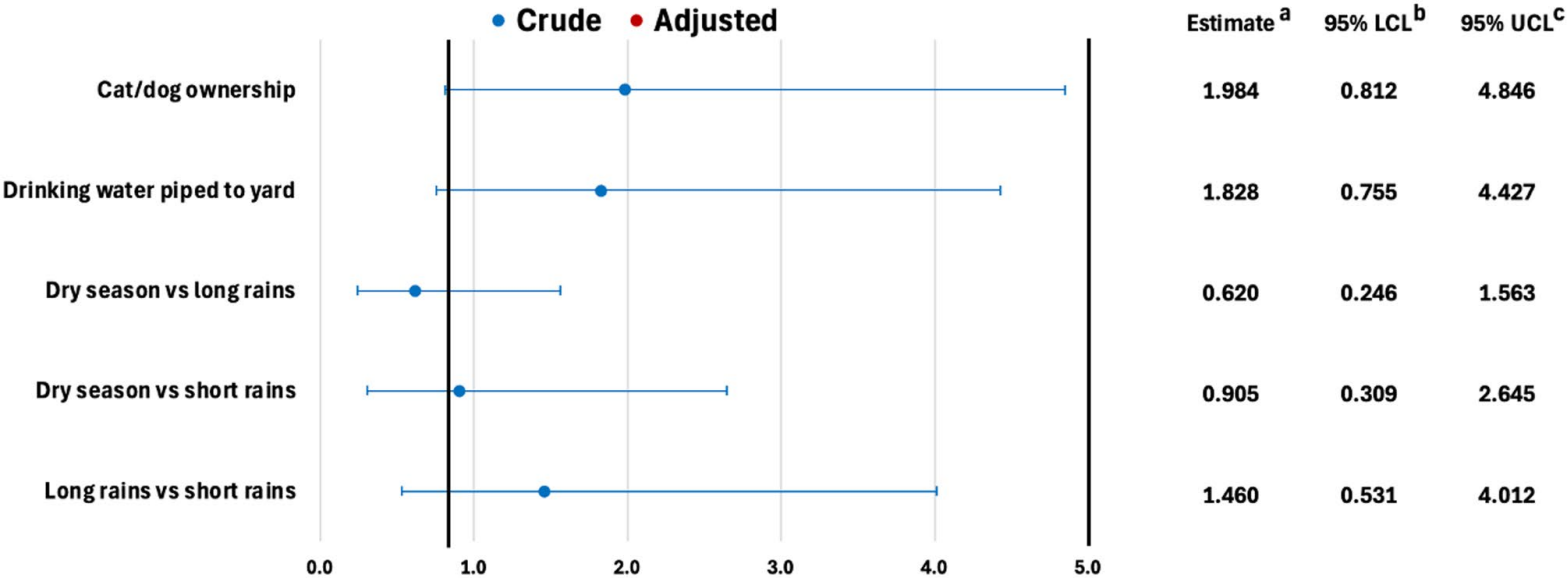
Crude and adjusted odds ratios for environmental exposures associated with diarrheal disease in Addis Ababa. ^a^ Odds ratio. ^b^ Wald lower confidence limit. ^c^ Wald upper confidence limit

In Addis Ababa, none of the considered environmental exposures were associated with diarrhea. Notably, there were insufficient households reporting exposure variables. Therefore, only cat or dog ownership, drinking water piped to yard, and season were considered in the analysis (Fig. 6). These exposures were assessed for confounding with household size; however, no adjusted estimates were reported since none of the exposures met confounder inclusion criteria.

**Fig. 7:**
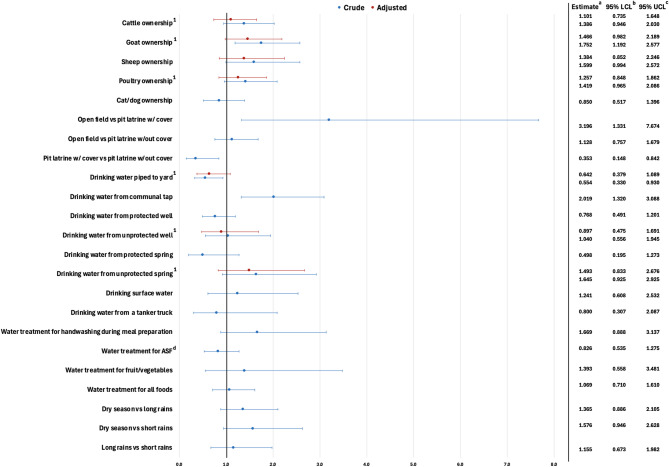
Crude and adjusted odds ratios for environmental exposures associated with diarrheal disease in Gondar. ^a^ Odds ratio. ^b^ Wald lower confidence limit. ^c^ Wald upper confidence limit. ^d^ Animal source foods. ^1^ Controlling for household size. ^2^ Controlling for household monthly income

In Gondar, only drinking water source and season were significantly associated with diarrhea (Fig. 7). Drinking water piped to the yard was protective for diarrhea in crude [OR: 0.575, (95% CI: 0.376, 0.881)] and adjusted [OR: 0.514, (95% CI: 0.331, 0.800)] analyses, and drinking water from an unprotected well [OR: 4.812, (95% CI: 2.026, 11.427)] was associated with diarrhea in crude analysis. Season of interview was associated with diarrhea with the odds of diarrhea decreasing in the dry season compared to the short rains season [OR: 0.424, (95%: 0.241, 0.747)] and the long rains season [OR: 0.546, (95%: 0.339, 0.878)]. Ownership of domesticated animals including cattle, goats, sheep, poultry, and cats or dogs was not significantly associated with diarrhea, even after adjusting for household size. Sanitation facility type was also not significantly associated, even after adjusting for household size and household monthly income. Drinking water from a communal tap, drinking water from a neighbor’s house, drinking water from a protected well, drinking water from a protected spring, and drinking water from an unprotected spring were not significantly associated with diarrhea. The use of treated or untreated water for the preparation of animal-source foods and the preparation of fruits and vegetables was not associated with diarrhea. Drinking water piped to the dwelling, surface water, and water from a tanker truck as well as water treatment for handwashing during meal preparation and for preparation of other foods (not animal or fruits/vegetables) were not tested due to low cell count.

**Fig. 8:**
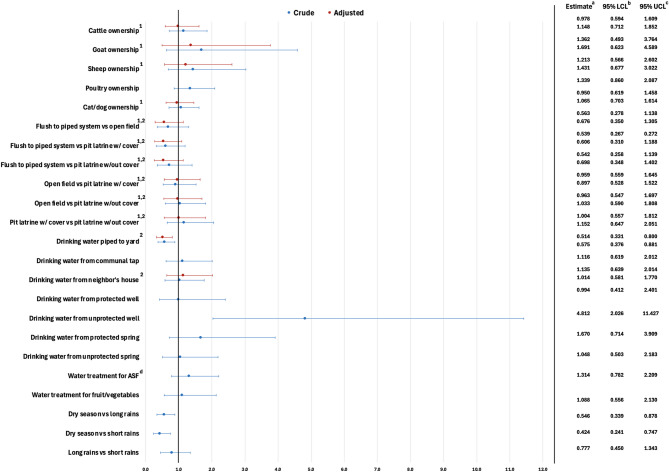
Crude and adjusted odds ratios for environmental exposures associated with diarrheal disease in Harar. ^a^ Odds ratio. ^b^ Wald lower confidence limit. ^c^ Wald upper confidence limit. ^d^ Animal source foods. ^1^ Controlling for household size

In Harar, goat ownership, sanitation facility type, and drinking water source were significantly associated with diarrhea (Fig. 8). Household goat ownership was significantly associated with diarrhea in the crude model but was not associated with diarrhea after controlling for household size. Additionally, household ownership of cattle, sheep, poultry, and cats or dogs was not associated with diarrhea in crude or adjusted models. The odds of diarrhea in households with open-field defecation were 3.196 times higher than in households reporting the use of a pit latrine with a cover. Additionally, the odds of diarrhea decreased for households using a pit latrine with a cover [OR: 0.353, (95% CI: 0.148, 0.842)] compared to households using a pit latrine without a cover. Due to low cell counts, piped to sewer/septic tank was not considered. Drinking water from a communal tap [OR: 2.019, (95%: 1.320, 3.088)] significantly increased the odds of diarrhea. Drinking water piped to the yard [OR: 0.554, (95%: 0.330, 0.930)] decreased the odds of diarrhea compared to households not consuming drinking water piped to the yard in the crude model but was no longer significant after adjusting for household size. Drinking water from a protected well, unprotected well, protected spring, unprotected spring, surface water, and tanker truck were not significantly associated with diarrhea in crude and adjusted models. Using treated water for meal preparation activities including handwashing, and preparing animal source foods, fruits and vegetables, and other foods was also not associated with diarrhea. Finally, season of interview was not statistically significant.

## Discussion

To the best of our knowledge, this study is the first to assess environmental risk factors for diarrhea at the community level in the Ethiopian general population, including adults and children, across multiple study sites. This study identified significant variation in environmental risk factors for household diarrhea by study site, which may be due to social, behavioral, infrastructural, and geographical differences between the study sites. For example, no environmental risk factors were associated with diarrhea in Addis Ababa, while multiple factors were identified in Gondar and Harar. However, these study sites have varying infrastructure and urbanicity. Previous research has estimated the burden of diarrheal disease in Ethiopia to be around 123,000 cases per 100,000 individuals, with children under five and the elderly impacted at higher rates [35]. Additionally, high rates of diarrhea can influence healthcare-seeking behavior, and childhood growth and development, resulting in costs to those with illness, their families, and the overall health system [36, 37]. Previous studies of children under the age of five in both southwest and northwest Ethiopia have identified sharing a household with domesticated animals, use of unimproved sanitation, and drinking water sources as factors associated with diarrhea [11, 12]. In our study, we found unimproved sanitation use, unimproved drinking water use, and season of interview to be associated with diarrhea, although these varied by study site. These results are similar to previous findings, suggesting environmental factors, especially those associated with water sources, contribute to diarrheal disease in Ethiopia.

Livestock ownership was largely not associated with diarrhea. This is consistent with a 2017 meta-analysis of livestock ownership and diarrhea in children that found that livestock ownership was not associated with diarrhea in Ethiopia, despite over 90% of households owning livestock [15]. In this study, only goat ownership was associated with diarrheal disease in the crude model in Harar, which is consistent with a previous study in the same location in Ethiopia [13] although this association was no longer significant after controlling for household size in our study. Interestingly, we did not find an association between diarrheal disease and ownership of other domesticated animals, which differs from a recent study conducted in rural households with children under the age of five in the same area [12]. This suggests that other household factors might confound the relationship between animal exposure and diarrhea, especially in predominantly rural areas. Given that we found an association between goat ownership and diarrhea in only one of three study sites, our results suggest that substantial variation exists in the relationship between domesticated animal ownership and diarrheal disease in Ethiopia, further work exploring pathogen-specific cases of diarrheal disease is needed.

Sanitation facility type was only associated with diarrhea in Harar. However, the association could not be tested in Addis Ababa due to the low number of households reporting diarrhea. The results from Harar in this study are similar to previous studies in predominantly rural areas reporting unimproved sanitation to be a risk factor for diarrhea in children. For example, a study of childhood diarrheal disease conducted using data from the 2016 Ethiopia Demographic and Health Survey identified use of unimproved sanitation, such as uncovered pit latrines, to be associated with diarrheal disease [19]. Additionally, a cross-sectional study conducted in a predominantly rural area of northwest Ethiopia of children under the age of five found that household latrine facility availability was associated with diarrheal disease [38]. Given that the association between sanitation facilities and diarrheal diseases varied by site in our study, there may be infrastructural, social, or resource availability differences across communities.

Consistent with previous studies in East Africa, drinking water from communal or unprotected sources was associated with diarrheal disease in Gondar and Harar. The use of uncovered drinking water or shared (community) drinking water has frequently been associated with diarrheal disease and these practices are common in Ethiopia with approximately one-third of the population using unimproved drinking water [23, 39]. A recent cross-sectional study conducted in Tigray, Ethiopia found that unimproved water sources were associated with increased diarrhea in children [7]. Our findings are also consistent with a study of diarrhea morbidity in children under the age of five in comparable countries. A study of children under the age of five using the Demographic and Health Survey data from Mali, Burkina Faso, Nigeria, and Niger found an association between use of unimproved drinking water sources, such as boreholes or tube wells, and diarrheal disease [40]. This indicates that interventions to improve drinking water practices are necessary, and efforts to address this issue should be community-specific due to the substantial geographic variation in findings. However, point-of-use water treatment practices like boiling or filtering have had limited use in past and little information regarding the efficacy of these methods in reducing diarrheal disease is available in Ethiopia [41]. Additional information regarding the feasibility of point-of-use water treatment is necessary to determine the efficacy of household water treatment.

Season of interview was associated with diarrhea in Gondar; however, the same association was not found in Addis Ababa and Harar. In Gondar, the dry season was associated with a decrease in the odds of diarrheal disease, which contradicts a 2017 retrospective cross-sectional study of children under the age of five in northwest Ethiopia which found the dry season to be the highest risk period for diarrheal disease [42]. Studies on the association between season and diarrheal diseases are limited in Ethiopia but have been considered in other regions with similar climates. For example, a previous study conducted in Indonesia found rainfall and rainy seasons to increase the odds of diarrhea, suggesting that climate can exacerbate environmental exposures [43]. Additionally, a study of children and adults with diarrhea in Senegal found that the prevalence of bacterial infections increased during the rainy season while viral infection in children under the age of five was more prevalent in the dry season [44]. Further, a study of diarrheagenic pathogens in Tanzania found that some pathogens, such as enteroaggregative *E. coli* and *Shigella* spp. were more prevalent during the dry season while others like *Giardia lamblia* were prevalent during the rainy season [45]. Given the variation in existing seasonality findings, further work exploring rainfall, temperature, and other climatic factors across different regions of Ethiopia is necessary to better understand the relationship.

There are several limitations to the present study that should be considered when interpreting results. First, diarrheal disease was self-reported, and we were not able to confirm whether diarrheal disease was due to enteric infection through stool sample testing. Second, risk factors were identified at the household level which may limit the ability to assess environmental risk factors for diarrhea. Third, exposures or behaviors of individuals with illness were not entirely captured, limiting our ability to identify all environmental risk factors for diarrheal disease. Fourth, due to the low number of illnesses in Addis Ababa, many environmental exposures were not assessed. Finally, this study was only conducted in three different regions of Ethiopia, which may not necessarily represent the entire country. However, efforts were taken to select three unique ecological zones to be most representative of Ethiopia.

## Conclusion

This cross-sectional study is, to our knowledge, the first to assess environmental risk factors associated with diarrheal diseases in both children and adults across multiple communities in Ethiopia. Multiple factors were associated with household diarrheal disease, including goat ownership, sanitation facility type, drinking water source, and season. These environmental factors can be managed through water treatment, improved sanitation practices, and educational programs focused on proper hygiene. For example, the use of unimproved sanitation and untreated water can be reduced through infrastructure changes, and interventions can be implemented to improve hygiene and promote proper contact with animals kept in or around the household. Improved WASH practices and containment and proper handling of livestock in the household like goats should be encouraged. Given the variation in risk factors by study site, exploration of geographical and infrastructural differences between sites should be considered when developing interventions to manage diarrheal disease within different communities in Ethiopia.

## Electronic supplementary material

Below is the link to the electronic supplementary material.

Supplementary Material 1

Supplementary Material 2

## Data Availability

The datasets generated and/or analyzed during the current study will be available in the Dryad repository, http://datadryad.org/stash/share/b5l5oLHJ0xy-g-ogS6wDCg202ipPvyfj-WCujChZmT0.

## Abbreviations

AOR: Adjusted odds ratio
COR: Crude odds ratio
DAGs: Directed acyclic graphs
FBD: Foodborne disease
IRB: Internal Review Board
LMICs: Low- and middle-income countries
WASH: Water, Sanitation, and Hygiene
WHO: The World Health Organization
