# Supplementary material for: Environmental risk factors associated with community diarrheal disease in Ethiopia
Source: BMC Public Health. 2025 May 27;25:1959. doi: 10.1186/s12889-025-23086-4 (PMC12107720; doi:10.1186/s12889-025-23086-4)
Supplement: Supplementary file 1 — Supplementary Material 1 [file 12889_2025_23086_MOESM1_ESM.docx]

**Additional File 1: Logistic regressions of environmental risk factors associated with diarrhea in Ethiopia, 2021-2022**

| **Addis Ababa** | | | | | |  |  |
| --- | --- | --- | --- | --- | --- | --- | --- |
| **Factor** | | **COR^a^ (95% CI^b^)** | **p-value** | **AOR^c^ (95% CI)** | **p-value** | **Confounders (% change in OR)** | **Goodness-of-fit (AOR)** |
| Cattle | Yes vs No | LCC^d^ | - |  |  |  |  |
| Goats | Yes vs No | LCC | - |  |  |  |  |
| Sheep | Yes vs No | LCC | - |  |  |  |  |
| Poultry | Yes vs No | LCC | - |  |  |  |  |
| Cats or dogs | Yes vs No | 1.984 (0.812, 4.846) | 0.1328 | - | - | NA | NA |
| Sanitation facility | No latrine facility/open field vs flush to piped sewer system or septic tank | LCC | - |  |  |  |  |
|  | Pit latrine with cover vs flush to piped sewer system or septic tank | LCC | - |  |  |  |  |
|  | Pit latrine without cover vs flush to piped sewer system or septic tank | LCC | - |  |  |  |  |
|  | No latrine facility/open field vs pit latrine with cover | LCC | - |  |  |  |  |
|  | No latrine facility/open field vs pit latrine without cover | LCC | - |  |  |  |  |
|  | Pit latrine with cover vs pit latrine without cover | LCC | - |  |  |  |  |
| Drinking water piped into dwelling | Yes vs No | LCC | - |  |  |  |  |
| Drinking water piped into yard | Yes vs No | 1.828 (0.755, 4.427) | 0.1813 | - | - | NA | NA |
| Drinking water from a communal tap | Yes vs No | LCC | - |  |  |  |  |
| Drinking water from a neighbor’s house | Yes vs No | LCC | - |  |  |  |  |
| Drinking water from a protected well | Yes vs No | LCC | - |  |  |  |  |
| Drinking water from an unprotected well | Yes vs No | LCC | - |  |  |  |  |
| Drinking water from a protected spring | Yes vs No | LCC | - |  |  |  |  |
| Drinking water from an unprotected spring | Yes vs No | LCC | - |  |  |  |  |
| Drinking water from surface water source | Yes vs No | LCC | - |  |  |  |  |
| Drinking water from a tanker truck | Yes vs No | LCC | - |  |  |  |  |
| Water treatment – handwashing^f^ | Yes vs No | LCC | - |  |  |  |  |
| Water treatment – ASF^g^ | Yes vs No | LCC | - |  |  |  |  |
| Water treatment – fruit/veg^h^ | Yes vs No | LCC | - |  |  |  |  |
| Water treatment – all other^i^ foods | Yes vs No | LCC | - |  |  |  |  |
| Season | Dry vs Long rains | 0.620 (0.246, 1.563) | 0.3108 | - | - | NA | NA |
|  | Dry vs Short rains | 0.905 (0.309, 2.645) | 0.8547 | - | - |  |  |
|  | Long rains vs Short rains | 1.460 (0.531, 4.012) | 0.4634 | - | - |  |  |
| **Gondar** | | | | | |  |  |
| **Factor** | | **COR^a^ (95% CI^b^)** | **p-value** | **AOR^c^ (95% CI)** | **p-value** | **Confounders (% change in OR)** | **Goodness-of-fit (AOR)** |
| Cattle | Yes vs No | 1.148 (0.712, 1.852) | 0.5716 | 0.978 (0.594, 1.609) | 0.9288 | Household size  (-14.8%) | 0.8379 |
| Goats | Yes vs No | 1.691 (0.623, 4.589) | 0.3022 | 1.362 (0.493, 3.764) | 0.5518 | Household size  (-19.5%) | 0.9725 |
| Sheep | Yes vs No | 1.431 (0.677, 3.022) | 0.3476 | 1.213 (0.566, 2.602) | 0.6198 | Household size  (-15.2%) | 0.3518 |
| Poultry | Yes vs No | 1.339 (0.860, 2.087) | 0.1966 | - | - | NA | NA |
| Cats or dogs | Yes vs No | 1.065 (0.703, 1.614) | 0.7666 | 0.950 (0.619, 1.458) | 0.8137 | Household size  (-10.8%) | 0.9284 |
| Sanitation facility | Flush to piped sewer system or septic tank vs No latrine facility/open field | 0.676 (0.350, 1.305) | 0.2437 | 0.563 (0.278, 1.138) | 0.1095 | Household size and Household monthly income (-16.7%%) | 0.2774 |
|  | Flush to piped sewer system or septic tank vs Pit latrine with cover | 0.606 (0.310, 1.188) | 0.1447 | 0.539 (0.267, 1.091) | 0.0858 | Household size and Household monthly income (-11.1%) |  |
|  | Flush to piped sewer system or septic tank vs Pit latrine without cover | 0.698 (0.348, 1.402) | 0.3126 | 0.542 (0.258, 1.139) | 0.1061 | Household size and Household monthly income (-22.3%) |  |
|  | No latrine facility/open field vs Pit latrine with cover | 0.897 (0.528, 1.522) | 0.6858 | 0.959 (0.559, 1.645) | 0.8787 | Household size and Household monthly income (6.5%) |  |
|  | No latrine facility/open field vs Pit latrine without cover | 1.033 (0.590, 1.808) | 0.9106 | 0.963 (0.547, 1.697) | 0.8965 | Household size and Household monthly income (-6.8%) |  |
|  | Pit latrine with cover vs Pit latrine without cover | 1.152 (0.647, 2.051) | 0.6315 | 1.004 (0.557, 1.812) | 0.9882 | Household size and Household monthly income (-12.8%) |  |
| Drinking water piped into dwelling | Yes vs No | LCC | - |  |  |  |  |
| Drinking water piped into yard | Yes vs No | 0.575 (0.376, 0.881) | 0.0109 | 0.514 (0.331, 0.800) | 0.0032 | Household monthly income (-10.6%) | 0.7435 |
| Drinking water from a communal tap | Yes vs No | 1.116 (0.619, 2.012) | 0.7146 | - | - | NA | NA |
| Drinking water from a neighbor’s house | Yes vs No | 1.014 (0.581, 1.770) | 0.9618 | 1.135 (0.639, 2.014) | 0.6659 | Household monthly income (10.7%) | 0.9361 |
| Drinking water from a protected well | Yes vs No | 0.994 (0.412, 2.401) | 0.9894 | - | - | NA | NA |
| Drinking water from an unprotected well | Yes vs No | 4.812 (2.026, 11.427) | 0.0004 | - | - | NA | NA |
| Drinking water from a protected spring | Yes vs No | 1.670 (0.714, 3.909) | 0.2370 | - | - | NA | NA |
| Drinking water from an unprotected spring | Yes vs No | 1.048 (0.503, 2.183) | 0.8995 | - | - | NA | NA |
| Drinking water from surface water source | Yes vs No | LCC | - |  |  |  |  |
| Drinking water from a tanker truck | Yes vs No | LCC | - |  |  |  |  |
| Water treatment – handwashing^f^ | Yes vs No | LCC | - |  |  |  |  |
| Water treatment – ASF^g^ | Yes vs No | 1.314 (0.782, 2.209) | 0.3024 | - | - | NA | NA |
| Water treatment – fruit/veg^h^ | Yes vs No | 1.088 (0.556, 2.130) | 0.8059 | - | - | NA | NA |
| Water treatment – all other^i^ foods | Yes vs No | LCC | - |  |  |  |  |
| Season | Dry vs Long rains | 0.546 (0.339, 0.878) | 0.0126 | - | - | NA | NA |
|  | Dry vs Short rains | 0.424 (0.241, 0.747) | 0.0030 | - | - |  |  |
|  | Long rains vs Short rains | 0.777 (0.450, 1.343) | 0.3666 | - | - |  |  |
| **Harar** | | | | | |  |  |
| **Factor** | | **COR^a^ (95% CI^b^)** | **p-value** | **AOR^c^ (95% CI)** | **p-value** | **Confounders (% change in OR)** | **Goodness-of-fit (AOR)** |
| Cattle | Yes vs No | 1.386 (0.946, 2.030) | 0.0939 | 1.101 (0.735, 1.648) | 0.6404 | Household size  (-20.6%) | 0.9953 |
| Goats | Yes vs No | 1.752 (1.192, 2.577) | 0.0043 | 1.466 (0.982, 2.189) | 0.0616 | Household size  (-16.3%) | 0.9559 |
| Sheep | Yes vs No | 1.599 (0.994, 2.572) | 0.0532 | 1.384 (0.852, 2.246) | 0.1890 | Household size  (-13.4%) | 0.7528 |
| Poultry | Yes vs No | 1.419 (0.965, 2.086) | 0.0752 | 1.257 (0.848, 1.862) | 0.2550 | Household size  (-11.4%) | 0.8528 |
| Cats or dogs | Yes vs No | 0.850 (0.517, 1.396) | 0.5206 | - | - | NA | NA |
| Sanitation facility^j^ | No latrine facility/open field vs flush to piped sewer system or septic tank | LCC | - |  |  |  |  |
|  | Pit latrine with cover vs flush to piped sewer system or septic tank | LCC | - |  |  |  |  |
|  | Pit latrine without cover vs flush to piped sewer system or septic tank | LCC | - |  |  |  |  |
|  | No latrine facility/open field vs pit latrine with cover | 3.196 (1.331, 7.674) | 0.0093 | - | - | NA | NA |
|  | No latrine facility/open field vs pit latrine without cover | 1.128 (0.757, 1.679) | 0.5537 | - | - |  |  |
|  | Pit latrine with cover vs pit latrine without cover | 0.353 (0.148, 0.842) | 0.0189 | - | - |  |  |
| Drinking water piped into dwelling | Yes vs No | LCC | - |  |  |  |  |
| Drinking water piped into yard | Yes vs No | 0.554 (0.330, 0.930) | 0.0254 | 0.642 (0.379, 1.089) | 0.1002 | Household size (13.7%) | 0.7953 |
| Drinking water from a communal tap | Yes vs No | 2.019 (1.320, 3.088) | 0.0012 | - | - | NA | NA |
| Drinking water from a neighbor’s house | Yes vs No | LCC | - |  |  |  |  |
| Drinking water from a protected well | Yes vs No | 0.768 (0.491, 1.201) | 0.2467 | - | - | NA | NA |
| Drinking water from an unprotected well | Yes vs No | 1.040 (0.556, 1.945) | 0.9022 | 0.897 (0.475, 1.691) | 0.7358 | Household size  (-13.8%) | 0.9482 |
| Drinking water from a protected spring | Yes vs No | 0.498 (0.195, 1.273) | 0.1455 | - | - | NA | NA |
| Drinking water from an unprotected spring | Yes vs No | 1.645 (0.925, 2.926) | 0.0903 | 1.493 (0.833, 2.676) | 0.1780 | Household size  (-15.2%) | 0.1815 |
| Drinking water from surface water source | Yes vs No | 1.241 (0.608, 2.532) | 0.5535 | - | - | NA | NA |
| Drinking water from a tanker truck | Yes vs No | 0.800 (0.307, 2.087) | 0.6488 | - | - | NA | NA |
| Water treatment – handwashing^f^ | Yes vs No | 1.669 (0.888, 3.137) | 0.1114 | - | - | NA | NA |
| Water treatment – ASF^g^ | Yes vs No | 0.826 (0.535, 1.275) | 0.3884 | - | - | NA | NA |
| Water treatment – fruit/veg^h^ | Yes vs No | 1.393 (0.558, 3.481) | 0.4776 | - | - | NA | NA |
| Water treatment – all other^i^ foods | Yes vs No | 1.069 (0.710, 1.610) | 0.7497 | - | - | NA | NA |
| Season | Dry vs Long rains | 1.365 (0.886, 2.105) | 0.1587 | - | - | NA | NA |
|  | Dry vs Short rains | 1.576 (0.946, 2.628) | 0.0809 | - | - |  |  |
|  | Long rains vs Short rains | 1.155 (0.673, 1.982) | 0.6019 | - | - |  |  |

^d^ Low cell count

^j^ option ‘Flush to piped sewer system or septic tank’ excluded due to low cell count
