# Supplementary material for: Environmental risk factors associated with community diarrheal disease in Ethiopia
Source: BMC Public Health. 2025 May 27;25:1959. doi: 10.1186/s12889-025-23086-4 (PMC12107720; doi:10.1186/s12889-025-23086-4)
Supplement: Supplementary file 2 — Supplementary Material 2 [file 12889_2025_23086_MOESM2_ESM.docx]

**Additional File 2: Household environmental characteristics in three communities, Ethiopia, 2021-2022**

|  | | **N (%)** | | | |
| --- | --- | --- | --- | --- | --- |
| **Characteristic** | | **Total (n=2436)** | **Addis Ababa (n=812)** | **Gondar (n=812)** | **Harar (n=812)** |
| Animal ownership | Cattle  Goats  Sheep  Poultry^a^  Cats/dogs | 556 (22.82)  399 (16.38)  186 (7.64)  555 (22.78)  667 (27.38) | 7 (0.86)  1 (0.12)  4 (0.49)  22 (2.71)  136 (16.75) | 189 (23.28)  26 (3.20)  54 (6.65)  227 (27.96)  371 (45.69) | 360 (44.33)  372 (45.81)  128 (15.76)  306 (37.68)  160 (19.70) |
| Sanitation facility | Flush to piped sewer system  Flush to septic tank  Pit latrine with cover  Pit latrine without cover  No latrine facility/open field  Other  Don’t know | 130 (5.34)  322 (13.22)  423 (17.36)  969 (39.78)  584 (23.97)  6 (0.25)  2 (0.08) | 108 (13.30)  182 (22.41)  119 (14.66)  391 (48.15)  12 (1.48)  0 (0.00)  0 (0.00) | 17 (2.09)  134 (16.50)  208 (25.62)  188 (23.15)  259 (31.90)  6 (0.74)  0 (0.00) | 5 (0.62)  6 (0.74)  96 (11.82)  390 (48.03)  313 (38.55)  0 (0.00)  2 (0.25) |
| Water source^b^ | Piped into dwelling  Piped into yard  Communal tap  Neighbor’s house  Protected well  Unprotected well  Protected spring  Unprotected spring  Surface water^c^  Rainwater  Tanker truck  Bottled water  Filtered water  Other  Don’t know | 196 (8.05)  1072 (44.01)  439 (18.02)  165 (6.77)  274 (11.25)  106 (4.35)  100 (4.11)  146 (5.99)  63 (2.59)  6 (0.25)  47 (1.93)  19 (0.78)  6 (0.25)  0 (0.00)  3 (0.12) | 154 (18.97)  478 (58.87)  164 (20.20)  9 (1.11)  3 (0.37)  1 (0.12)  5 (0.62)  0 (0.00)  0 (0.00)  1 (0.12)  1 (0.12)  3 (0.37)  5 (0.62)  0 (0.00)  1 (0.12) | 12 (1.48)  407 (50.12)  110 (13.55)  134 (16.50)  48 (5.91)  23 (2.83)  37 (4.56)  69 (8.50)  8 (0.99)  2 (0.25)  7 (0.86)  2 (0.25)  0 (0.00)  0 (0.00)  1 (0.12) | 30 (3.69)  187 (23.03)  165 (20.32)  22 (2.71)  223 (27.46)  82 (10.10)  58 (7.14)  77 (9.48)  55 (6.77)  3 (0.37)  39 (4.80)  14 (1.72)  1 (0.12)  0 (0.00)  1 (0.12) |
| Use of treated water for handwashing during meal preparation | Yes  No  Don’t know^d^ | 372 (15.27)  2057 (84.44)  7 (0.29) | 201 (24.75)  609 (75.00)  2 (0.25) | 56 (6.90)  754 (92.86)  2 (0.25) | 115 (14.16)  694 (85.47)  3 (0.37) |
| Use of treated^e^ water to prepare animal source foods | Yes  No | 462 (18.97)  1974 (81.03) | 90 (11.08)  722 (88.92) | 138 (17.00)  674 (83.00) | 234 (28.82)  578 (71.18) |
| Use of treated^e^ water to prepare fruits or vegetables | Yes  No | 131 (5.38)  2305 (94.62) | 19 (2.34)  793 (97.66) | 82 (10.10)  730 (89.90) | 30 (3.69)  782 (96.31) |
| Use of treated^e^ water to prepare all other^f^ foods | Yes  No | 395 (16.22)  2041 (83.78) | 140 (17.24)  672 (82.76) | 5 (0.62)  807 (99.38) | 250 (30.79)  562 (69.21) |

^a^ Poultry includes chickens, ducks, other birds kept for consumption

^b^ Some households reported more than one water source

^c^ Rivers, lakes, canals, etc.

^d^ Excluded from analyses due to low cell count

^e^ boiled/filtered water

^f^ foods not including animal source products, fruits, and vegetables
